# A bibliometric and visualization analysis of glucocorticoid-induced osteoporosis research from 2012 to 2021

**DOI:** 10.3389/fendo.2022.961471

**Published:** 2022-08-05

**Authors:** Buchan Jiang, Chengyao Feng, Chenbei Li, Chao Tu, Zhihong Li

**Affiliations:** ^1^ Department of Orthopaedics, The Second Xiangya Hospital of Central South University, Changsha, China; ^2^ Hunan Key Laboratory of Tumor Models and Individualized Medicine, Changsha, China

**Keywords:** glucocorticoids, osteoporosis, bibliometrics, visualization, hotspots

## Abstract

**Introduction:**

Glucocorticoid-induced osteoporosis (GIOP) is the most common cause of secondary osteoporosis. Although many studies related to GIOP have been published, there was no bibliometric analysis in this field. This study aimed to investigate the research trends on GIOP by using bibliometric analysis.

**Materials and Methods:**

All data were collected from the Web of Science Core Collection (WoSCC). All original research articles regarding GIOP from 2012 to 2021 were retrieved. CiteSpace was used to analyze the distribution of countries, institutions, journals, authors, and keywords. We revealed hotspots and trends in the field by drawing co-occurrence keyword maps and identifying burst keywords.

**Results:**

From 2012 to 2021, 685 relevant articles were published, with a peak in 2018 in the annual number of publications. China and McMaster University were the leading country and institution in this field with 208 and 12 publications, respectively. Osteoporosis International was the journal with the most studies, while Journal of Bone and Mineral Research was the most cited journal. “Bone mineral density”, “fracture”, “postmenopausal women”, “prevention” and “therapy” were the most high-frequency keywords, while “bone mineral density”, “bisphosphonate” and “metabolism” were the top high-centrality keywords.

**Conclusion:**

The results from this bibliometric study provided insight into the status and research trends in GIOP of the past decade, which could help researchers quickly determine the current hotspots and frontier trends in this field.

## Introduction

Glucocorticoid (GC), such as dexamethasone and methylprednisolone, is widely used in the treatment of multiple inflammatory and autoimmune diseases due to its potent anti-inflammatory and immunosuppressive effects (1, 2). It is estimated that 1% to 2% of the world’s population is receiving long-term treatment with GC (3). However, long-term use of GC may lead to diabetes, glaucoma, osteoporosis, and other adverse events. Glucocorticoid-induced osteoporosis (GIOP) is one of the most severe side effects of GC, and the third most common form of osteoporosis after postmenopausal osteoporosis and senile osteoporosis but the most frequent cause of secondary osteoporosis (4). Like other types of osteoporosis, GIOP is characterized by decreased bone mass and microarchitectural deterioration of bone tissue, resulting in increased bone fragility (5). The damage of GC to the function and viability of osteoblasts is considered to be the primary mechanism of GC-induced bone loss (6). The major adverse clinical outcome of GIOP is fragility fractures, which could place a huge burden on patients and their families (7, 8). The research history of GIOP is decades long since the adverse effects of GC on bone was first recognized almost 90 years ago (9–11), and numerous clinical or basic studies related to GIOP have been reported.

To date, however, no bibliometric study focusing on GIOP research has been reported. Bibliometrics is a mathematical-statistical tool to gain insight into the current status, trends, and future directions of a specific research field through identifying and evaluating some quantitative factors like quantity of papers and geographical distributions (12–14). In addition, bibliometric analysis can also serve as a reference for the government to formulate specific policies, guide the funding and reward scientific researchers (15). Due to these advantages, bibliometric analysis has been widely conducted on various research topics in medical fields, including osteoporosis (16), postmenopausal osteoporosis (15), male osteoporosis (17), etc. Based on the research of GIOP in recent years, it is necessary to conduct the first bibliometric analysis on this topic to present the face of this research field.

In this study, we conducted a bibliometric analysis to systematically analyze the trends, hotspots, and new frontiers of GIOP research in the past decade based on the bibliometric software CiteSpace, so as to make it accessible to comprehensively understand the research background and development in this field.

## Materials and methods

### Data collection and search strategy

Relevant literature was collected from the Web of Science Core Collection (WoSCC). Three indexes, the Science Citation Index Expanded (SCI-Expanded); the Social Sciences Citation Index (SSCI); and the Emerging Sources Citation Index (ESCI), were selected from the WoSCC as the data source. The search was performed on March 19, 2022. The publication type was limited to “article” with language restriction to English. To reflect the current state of GIOP research, we retrieved the articles that published in the recent decade, and the time interval was set from January 1, 2012 to December 31, 2021. The detailed search strategies are presented in **Table 1**.** A** total of 685 papers were finally identified. “Full Record and Cited References” of these records including titles, authors, abstracts, and cited references were exported in plain text format.

**Table 1:** Summary of data selection strategy in this study.

Content			
Data source	Web of Science Core Collection		
Time span	2012-2021		
Languages	English		
Literature types	Article		
Search strategy	#1	2537	[TS=(“glucocorticoid* induced osteoporosis”) OR TS=(“steroid* induced osteoporosis”) OR TS=(“corticosteroid* induced osteoporosis”) OR TS=(“corticoid* induced osteoporosis”) OR TS=(“dexamethasone induced osteoporosis”) OR TS=(“prednisone-induced osteoporosis”) OR TS=(“prednisolone-induced osteoporosis”) OR TS=(“methylprednisolone-induced osteoporosis”)OR TS=(“glucocorticoid* osteoporosis”) OR TS=(“steroid* osteoporosis”) OR TS=(“corticosteroid* osteoporosis”) OR TS=(“corticoid* osteoporosis”)]
	#2	685	#1 AND DT=(“ARTICLE”) AND LA=(“ENGLISH”) AND PY=(2012-2021)

### Research tools

All valid data were imported to Microsoft Excel 2019 and CiteSpace (5.8R3) for performing visual analysis. Microsoft Office Excel 2019 was used to analyze the trend of the number of articles published by year. CiteSpace, which is a java-based information visualization software developed by Dr. Chaomei Chen (School of Information Science and Technology, Drexel University, Philadelphia, PA, USA) (18), was utilized to visually analyze countries, institutions, authors, journals, cited references, keywords, as well as keywords with strong citation bursts over time. Related visualization knowledge maps which consist of nodes and links were drawn. In these maps, the nodes represent countries, institutions, authors, journals, cited references, etc. The links represent the cooperation, co-occurrence, or co-citation relationships between two nodes. The bigger the size of a node, the greater occurrence or citation frequency of the node. The color of nodes indicates the occurrence or citation years. Centrality is an index for quantitatively evaluating the importance of a node in a network, and a centrality greater than 0.1 was considered significant. A node with outer purple trim indicates high centrality, and the thickness of the purple trim represents the size of centrality.

## Results

### Publication years

**Figure 1:**
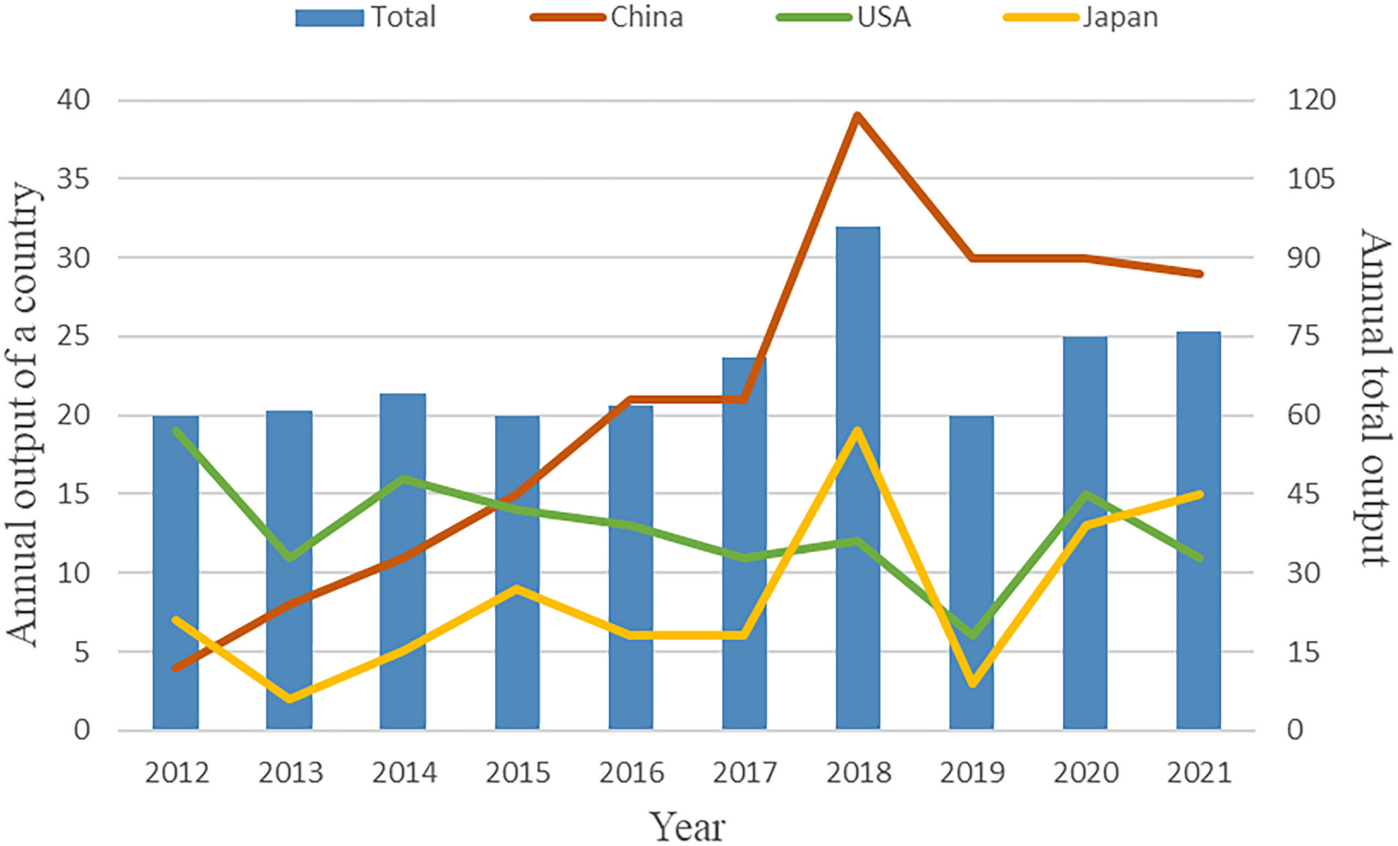
The number of annual publications.

A total of 685 publications were found after removing duplications. As shown in **Figure 1**, the number trend of annual global publications related to GIOP remained relatively steady overall in the past decade, with 60 or more publications per year. However, there was a sudden and noticeable spike in 2018 with 96 published articles. As for the annual output of the three most productive countries (China, the USA and Japan, which will be illustrated below), China showed the most significant overall growth, ranking first in the annual output since 2015 and reaching its peak in 2018. The number trend of Japanese publications also showed an overall increase in the past decade with a peak in 2018. In contrast, this trend of the USA was fluctuating. These results indicate that the research trend of GIOP varies greatly among different countries.

### Analysis of countries and institutions

**Figure 2:**
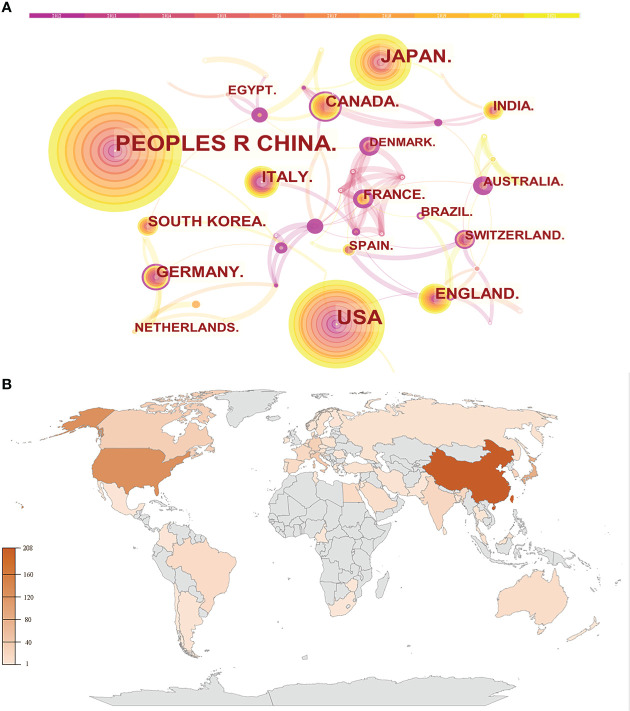
Visualization of countries. **(A)** Collaboration network of countries. **(B)** World map of publications distributed in various countries.

As shown in **Figure 2A**, the country distribution map consists of 58 nodes and 94 links. Besides, a detailed overview has been presented as a world map (**Figure 2B**). The top 5 countries with the highest number of publications were China, the USA, Japan, Italy, and UK, while Sweden, Saudi Arabia, Australia, Denmark, and Malaysia were the top 5 countries in terms of centrality (**Table 2**). China was the most productive country with 208 publications, followed by the USA with 128 papers; Sweden had the highest centrality at 0.67, and this was followed by Saudi Arabia at 0.41. Interestingly, none of the top 5 productive countries ranked in the top 10 in terms of centrality, indicating that the global influence of these countries was not proportional to their quantity.

**Table 2:** Top 10 countries in terms of publications and centrality.

Rank	Publications (% of 685)	Country	Rank	Centrality	Country
1	208 (30.4%)	China	1	0.67	Sweden
2	128 (18.7%)	USA	2	0.41	Saudi Arabia
3	85 (12.4%)	Japan	3	0.4	Australia
4	43 (6.3%)	Italy	4	0.29	Denmark
5	41 (6.0%)	UK	5	0.27	Malaysia
6	36 (5.3%)	Canada	6	0.23	Sri Lanka
7	34 (5.0%)	Germany	7	0.22	France
8	27 (3.9%)	South Korea	8	0.18	Argentina
9	24 (3.5%)	India	9	0.18	Belgium
10	22 (3.2%)	France	10	0.16	Austria

**Figure 3:**
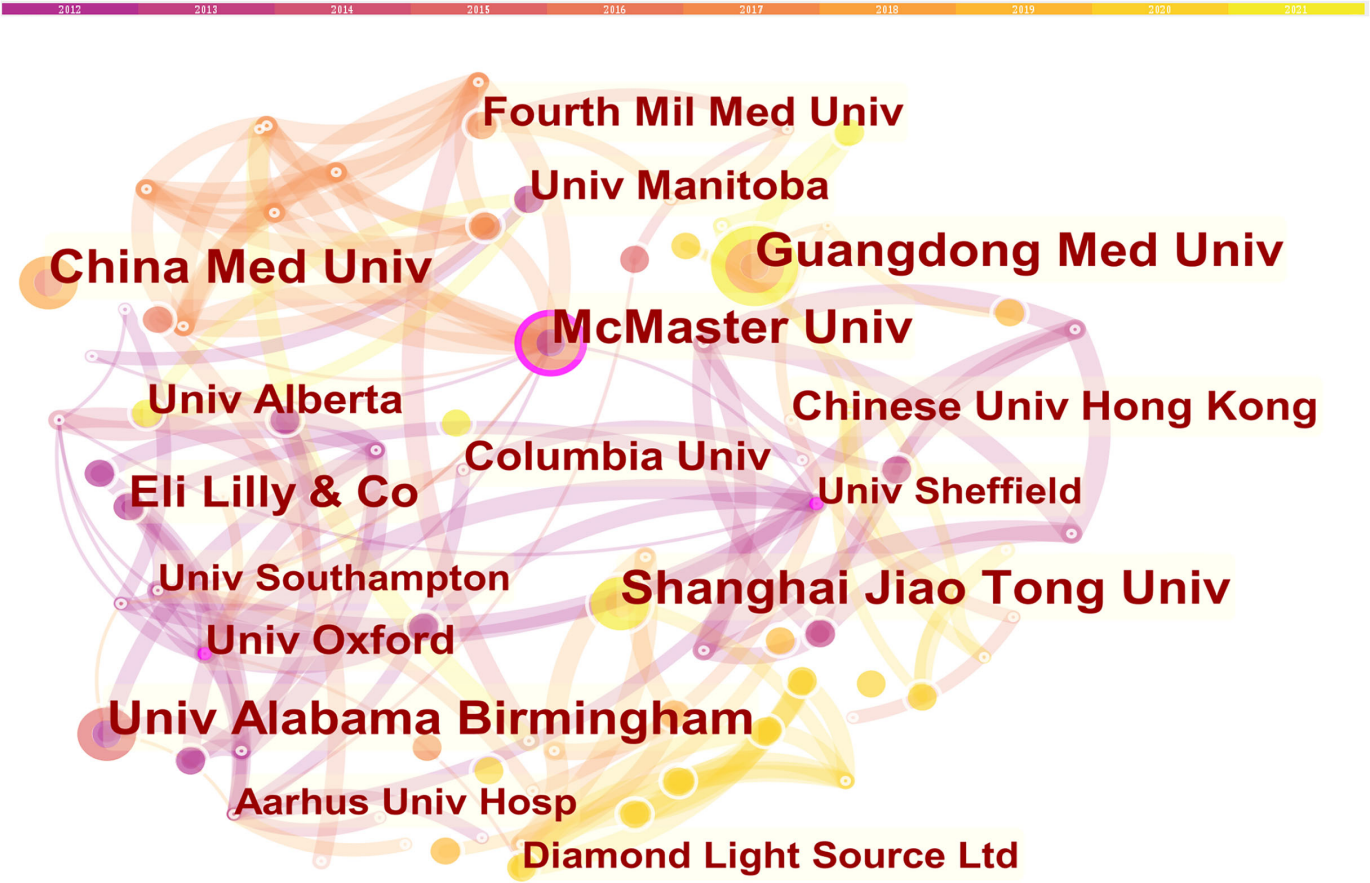
Collaboration network of institutions.

The co-institution network map is shown in **Figure 3**, with 309 nodes and 660 links. The top 3 prolific institutions were McMaster University, Shanghai Jiao Tong University and China Medical University (**Table 3**). In terms of centrality, McMaster University, University of Sheffield, and University of Oxford were the top 3 institutions. McMaster University had both the most publications with 12 papers and the highest centrality at 0.17.

**Table 3:** Top 10 institutions in terms of publication volume and centrality.

Rank	Institution	Country	Publications	Rank	Institution	Country	Centrality
1	McMaster University	Canada	12	1	McMaster University	Canada	0.17
2	Shanghai Jiao Tong University	China	11	2	University of Sheffield	UK	0.14
3	China Medical University	China	11	3	University of Oxford	UK	0.12
4	University of Alabama at Birmingham	USA	10	4	Mayo Clinic	USA	0.09
5	Guangdong Medical University	China	10	5	Chinese University of Hong Kong	China	0.08
6	Eli Lilly and Company	USA	9	6	Centre Hospitalier Universitaire de Nancy	France	0.08
7	University of Oxford	UK	8	7	Diamond Light Source Ltd	UK	0.07
8	University of Alberta	Canada	8	8	University of Western Ontario	Canada	0.07
9	Fourth Military Medical University	China	8	9	Aarhus University Hospital	Denmark	0.06
10	University of Manitoba	Canada	8	10	University of Alabama at Birmingham	USA	0.05

### Analysis of authors

**Figure 4:**
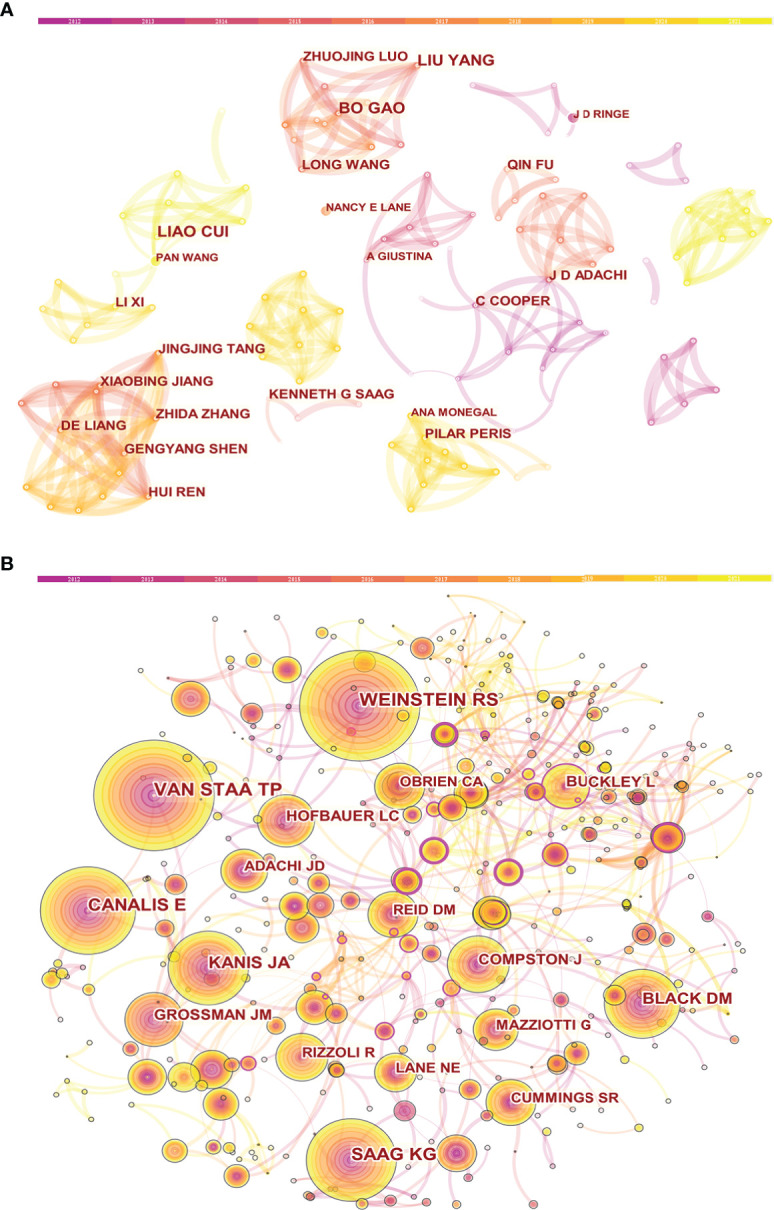
Visual analysis of authors. **(A)** Collaboration network of co-authors. **(B)** Network visualization map of cited authors.

The co-authorship and cited authors were analyzed to identify potential partnerships. The co-authorship network (**Figure 4A**) was composed of 380 nodes and 785 links. Among the authors, Liu Yang from China was the author who had the highest number of papers (n=10), and this was followed by Kenneth G. Saag (n=9) from the USA (**Table 4**). It is worth noting that none of the authors had a significant centrality, reflecting the lack of cooperation among them. **Figure 4B** displays the network of cited authors, with 490 nodes and 739 links. Robert S. Weinstein from the USA had the highest citation counts (n=223), followed by Tjeerd Pieter van Staa (n=216) from Netherlands and Ernesto Canalis (n=176) from the USA (**Table 4**).

**Table 4:** The top 5 prolific authors and cited authors.

Rank	Author	Institution (Country)	Publications	Centrality		Rank	Cited Author	Institution (Country)	Citation Counts	Centrality
1	Liu Yang	Fourth Military Medical University (China)	10		0.01	1	Robert S Weinstein	University of Arkansas for Medical Sciences (USA)	223	0.01
2	Kenneth G Saag	University of Alabama at Birmingham (USA)	9		0.03	2	Tjeerd Pieter van Staa	University of Utrecht (Netherlands)	216	0.04
3	Jonathan D Adachi	McMaster University (Canada)	8		0.05	3	Ernesto Canalis	University of Connecticut Health Center (USA)	176	0.01
4	Andrea Giustina	San Raffaele Vita Salute University (Italy)	8		0.01	4	Kenneth G Saag	University of Alabama at Birmingham (USA)	150	0.01
5	Lorenz C Hofbauer	Dresden University of Technology (Germany)	8		0.02	5	John A Kanis	University of Sheffield (UK)	123	0.02

### Analysis of journals

The 685 papers were published in 346 journals. Journal Citation Reports 2020 was used to obtain the impact factor (IF) and quartile (Q) of a journal category. The top 10 journals in terms of publication volume and citation counts are shown in **Table 5** with their IF and quartile in category. *Osteoporosis International* was the most productive journal with 44 papers published, which was followed by *Bone* with 28 publications. Among the 10 journals, *Journal of Bone and Mineral Research* was the journal with the highest impact factor (IF), with an IF of 6.741. The journal co-citation analysis was also conducted to reveal the interdependence and cross-relationship among journals. The top-ranked journal by citation counts was *Journal of Bone and Mineral Research* with 529 citations, followed by *Osteoporosis International* (488 citations) and *Bone* (471 citations). Among the 10 top-cited journals, *New England Journal of Medicine* had the highest IF of 91.253.

**Table 5:** The top 10 journals distributed by publications and citations.

Rank	Journal	Article Counts	IF (2020)	Quartile in Category (2020)	Rank	Cited Journal	Co-Citation Counts	IF (2020)	Quartile in Category (2020)
1	Osteoporosis International	44	4.507	Q2	1	Journal of Bone and Mineral Research	529	6.741	Q1
2	Bone	28	4.398	Q2	2	Osteoporosis International	488	4.507	Q2
3	Journal of Bone and Mineral Metabolism	17	2.626	Q3/Q4	3	Bone	471	4.398	Q2
4	Journal of Bone and Mineral Research	11	6.741	Q1	4	New England Journal of Medicine	325	91.253	Q1
5	Journal of Clinical Endocrinology & Metabolism	11	5.958	Q1	5	Journal of Clinical Endocrinology & Metabolism	305	5.958	Q1
6	Molecular Medicine Reports	11	2.952	Q3/Q4	6	Calcified Tissue International	279	4.333	Q2
7	PLOS ONE	11	3.24	Q2	7	Arthritis & Rheumatology	229	10.995	Q1
8	Calcified Tissue International	9	4.333	Q2	8	Journal of Clinical Investigation	222	14.808	Q1
9	Scientific Reports	9	4.38	Q1	9	Endocrinology	215	4.736	Q2
10	Archives of Osteoporosis	8	2.617	Q2/Q4	10	Journal of Bone and Mineral Metabolism	195	2.626	Q3/Q4

### Analysis of co-cited references

**Table 6** demonstrates the top 5 most co-cited references. They were co-cited more than 25 times, of which the most frequently cited one was titled *American College of Rheumatology 2010 Recommendations for the Prevention and Treatment of Glucocorticoid-Induced Osteoporosis* and published in *Arthritis Care & Research* (IF=4.794). The second top cited paper was titled *Glucocorticoid-Induced Bone Disease* and published in *New England Journal of Medicine* (IF=91.253, which is the leading journal in clinical medicine). These 5 references could be considered as the most popular papers in this field.

**Table 6:** The top 5 cited references.

Rank	Title	Cited Frequency	Year	First Author	Journal	IF (2020)	Quartile in Category (2020)	
1	American College of Rheumatology 2010 Recommendations for the Prevention and Treatment of Glucocorticoid-Induced Osteoporosis	43	2010	Jennifer M Grossman	Arthritis Care & Research	4.794	Q2
2	Glucocorticoid-Induced Bone Disease	33	2011	Robert S Weinstein	New England Journal of Medicine	91.253	Q1
3	2017 American College of Rheumatology Guideline for the Prevention and Treatment of Glucocorticoid-Induced Osteoporosis	31	2017	Lenore Buckley	Arthritis & Rheumatology	10.995	Q1
4	A framework for the development of guidelines for the management of glucocorticoid-induced osteoporosis	31	2012	Sarath Lekamwasam	Osteoporosis International	4.507	Q2
5	Glucocorticoid-induced osteoporosis: an update	26	2018	Juliet Compston	Endocrine	3.633	Q3

### Analysis of keywords

**Figure 5:**
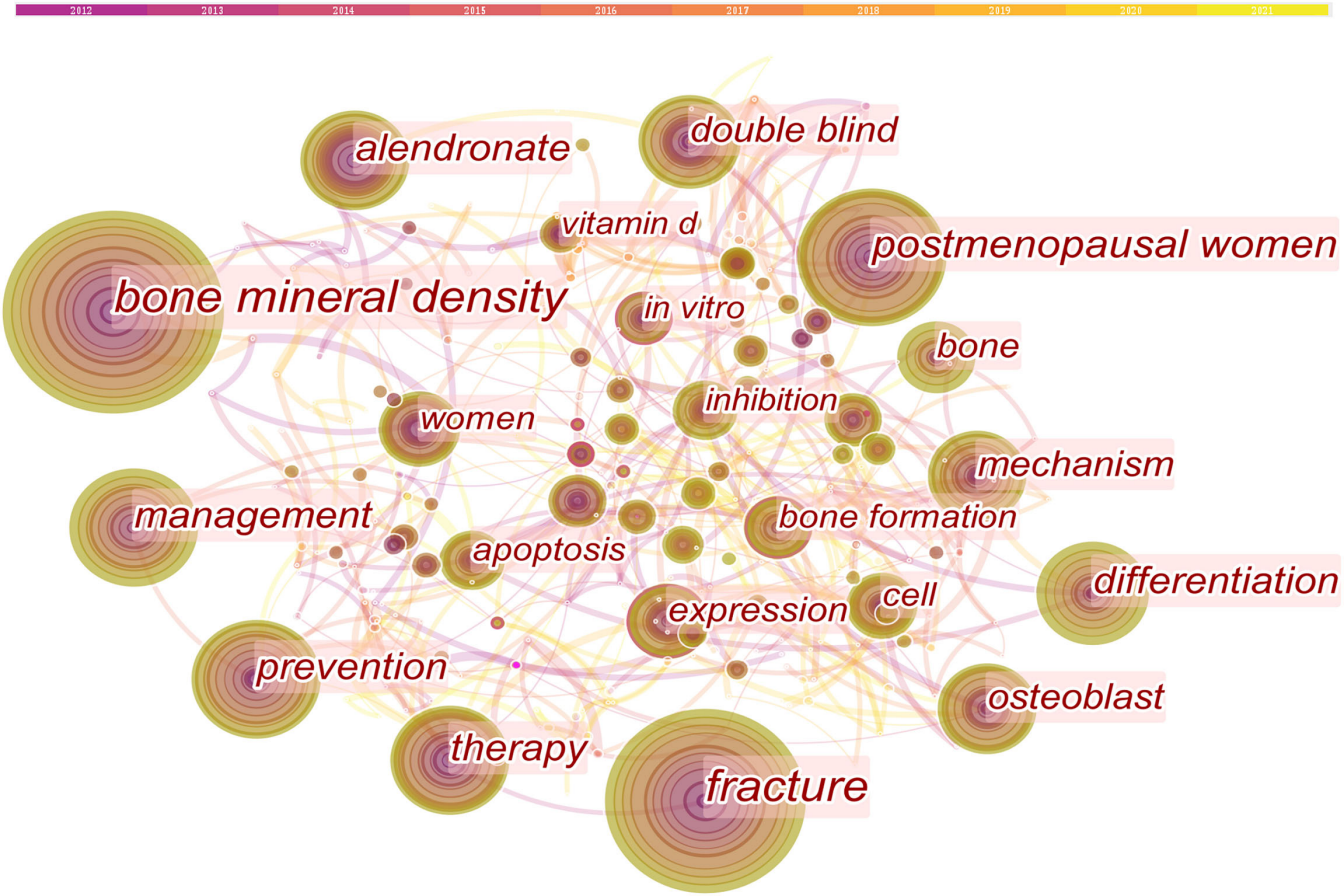
Co-occurring keywords map.

The keywords with a high frequency represent hot topics, while high-centrality keywords reflect the influence of corresponding research content in a certain field. The keywords co-occurrence is illustrated in **Figure 5** which consists of 396 nodes and 651 links. **Table 7** demonstrates that the top 10 high-frequency keywords on this topic were bone mineral density, fracture, postmenopausal women, prevention, therapy, management, differentiation, alendronate, double blind, and Mechanism. The top 10 high-centrality keywords were bone mineral density, bisphosphonate, metabolism, rheumatoid arthritis, osteoclastogenesis, guideline, microarchitecture, trabecular bone score, bone formation, and adipogenesis.

**Table 7:** The top 10 keywords in terms of frequency and centrality.

Rank	Frequency	Keywords	Rank	Centrality	Keywords
1	213	Bone mineral density	1	0.27	Bone mineral density
2	169	Fracture	2	0.26	Bisphosphonate
3	105	Postmenopausal women	3	0.21	Metabolism
4	89	Prevention	4	0.17	Rheumatoid arthritis
5	78	Therapy	5	0.15	Osteoclastogenesis
6	78	Management	6	0.15	Guideline
7	76	Differentiation	7	0.14	Microarchitecture
8	75	Alendronate	8	0.14	Trabecular bone score
9	71	Double blind	9	0.13	Bone formation
10	68	Mechanism	10	0.12	Adipogenesis

**Figure 6:**
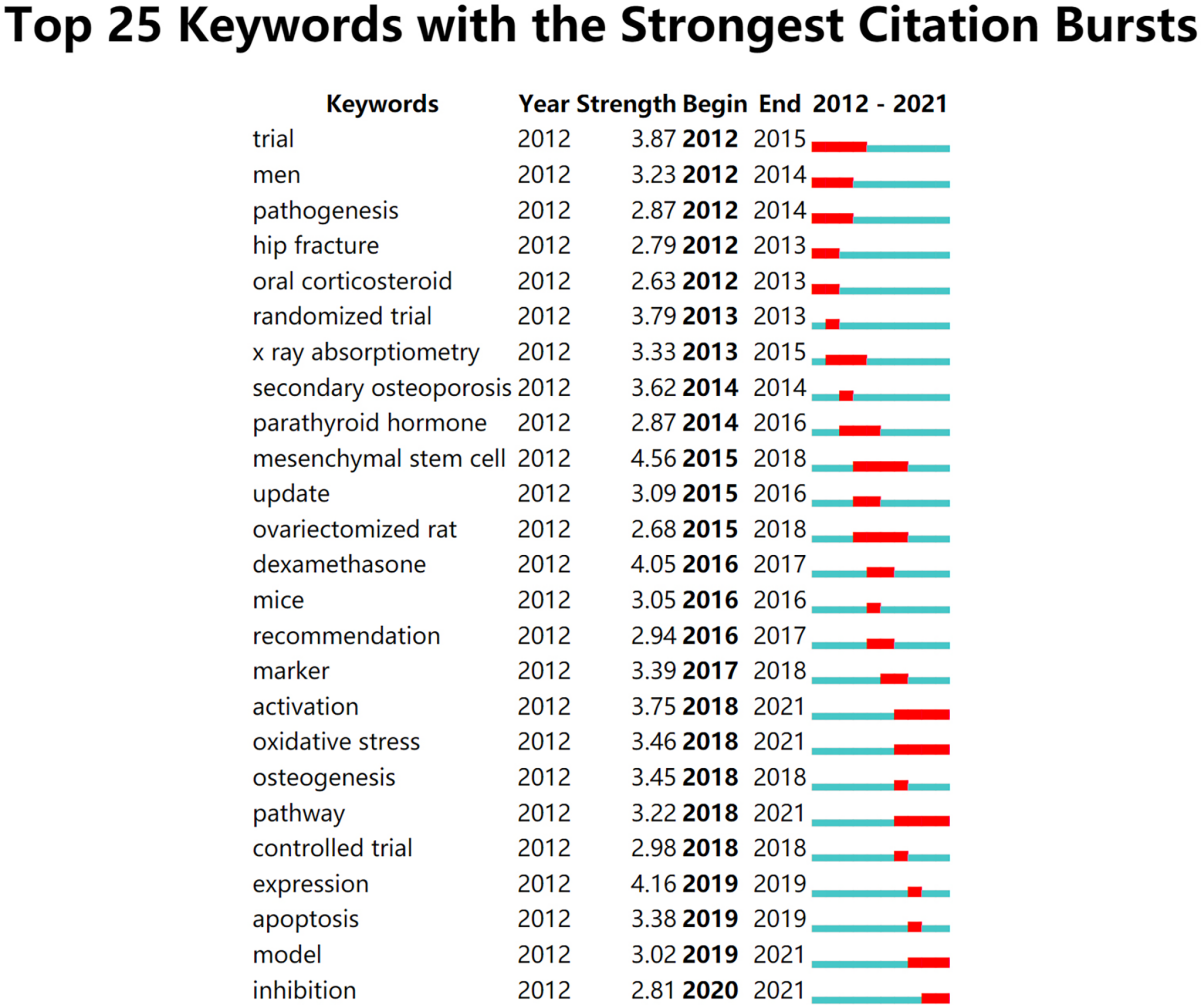
Top 25 keywords with the strongest citation bursts.

A citation burst refers to the increasing citation within a certain time interval, which could reflect the development of cutting-edge research topics (19). **Figure 6** has shown the top 25 keywords with the strongest citation burst from 2012 to 2021. The keyword “mesenchymal stem cell” which appeared in 2015 was the keyword with the strongest citation burst. There were 5 burst keywords that continued to 2021 (activation, oxidative stress, pathway, model, inhibition).

## Discussion

### General information

To our knowledge, this is the first bibliometric and visualized analysis of GIOP. In this analysis, a total of 685 original articles published from 2012 to 2021 were acquired from the Web of Science Core Collection. From the dynamic change in the number of annual total publications, we observed that the research trend on this domain remained relatively stable in recent years. The total annual output saw a significant rise and peak in around 2018, although it subsequently fell back to its previous level. This rise and peak were mainly contributed by some countries especially China. China witnessed an exponential increase in the number of annual publications before 2018, suggesting the rapidly growing attention paid by experts and scholars on this topic, and this number has fallen afterwards but still remains at its plateau. These results indicate that the research on GIOP in China might have reached its peak in the past decade. In contrast, the USA had a fluctuating annual output in the recent decade, making it hard to predict its future trend. According the total annual output of the world in recent three years, it can be estimated that the publication amount in this field will remain relatively stable in the coming years.

Analyzing the literature sources, 58 countries and 309 institutes published these 685 papers. China, holding the highest publication volume, also accounts for three of the top 5 most productive institutions. Therefore, China was the most prolific and active country, which was followed by the USA and Japan, and these three countries together published more than 60% of these 685 papers. Although some countries especially Sweden, Saudi Arabia and Australia did not have a great number of papers, they were the top countries when in terms of centrality, indicating the quality of their papers is very high. As for research institutions, McMaster University was both the most productive and most high-centrality one, suggesting its dominating position in this research field. It is worth noting that apart from that of China, all the other institutions in **Table 3** are in developed countries, especially Canada, UK, and the USA, reflecting their huge academic influence in this field. Meanwhile, close cooperation can be observed between different countries and institutions in **Figure 2A** and **Figure 3**, which would promote the convergence and development of knowledge in this field.

From the perspective of authors, 380 authors contributed to these 685 papers. The author with the most publications was Liu Yang from the Fourth Military Medical University, Xi’an, China. Her research has long focused on bone marrow-derived mesenchymal stem cells (BMSCs) and osteoporosis. In the recent decade, her team has published several papers about the therapeutic effects of some bioactive components extracted from Chinese medicinal herbs on GIOP. For instance, one study discovered that the administration of tetramethylpyrazine prevented bone mass decrease in GIOP rats by prolonging BMSC survival (20). In another basic study, her team revealed that GC could promote osteoclast formation *via* autophagy, providing support for autophagy as a therapeutic target in GIOP (21). We also found that the most cited author was Robert S. Weinstein, an endocrinologist from the University of Arkansas for Medical Sciences, Little Rock, Arkansas, USA. His studies mainly focused on the basic research on GC-induced bone diseases. For example, one of his studies reported the positive effects of GC on osteoclast survival *in vitro*, which later attracted the researchers’ attention to the mechanism of GC on regulating osteoclastic bone resorption (22).

In the journal distribution analysis, the top 10 productive journals in **Table 5** are considered to be the core journals for GIOP. In addition, most of these journals belong to Q1 or Q2, indicating that the research on GIOP is greatly valued in the global scientific field. In the future, more GIOP-related studies will be published in these journals. **Table 5** also lists the top 10 journals with the largest citations, which means that they have published high-quality studies that captured the attention of scholars who were interested in this field. It is worth noting that *Osteoporosis International* and *Bone* were both in the top 3 journals in terms of productivity and co-citations, indicating their great impact in this field. Totally, our results would be helpful for researchers in this field to quickly find appropriate journals to obtain the latest advances regarding GIOP research or submit their articles.

According to the analysis of references, the top 5 cited references were published between 2010 and 2018 and all of them were focusing on GIOP. The most frequently cited reference was authored by Jennifer M Grossman et al. (23). This paper was recommendations for counseling and monitoring GIOP offered by American College of Rheumatology, providing a guideline for the management of GIOP. The second most cited one was authored by Robert S Weinstein (24), in which he reviewed the experience of clinical practice in the treatment of glucocorticoid-induced bone disease. The third most cited one was authored by Lenore Buckley et al. (25), which was a guideline for the prevention and treatment of GIOP. It is interesting that the document type of all these 5 papers is review, indicating that they nicely summarized the existing achievements and provided a good guidance in this field. They are thought to be milestones in this field, which will serve as the foundation for future studies.

### Hotspots in GIOP-related research in the recent decade

A keyword reflects the research theme of a paper. Therefore, research hotspots can be known by analyzing the frequency of keywords in a certain field. In this analysis, the top 3 frequent keywords were “bone mineral density”, “fracture” and “postmenopausal women”. The burst keywords detection found that “mesenchymal stem cell” had the highest burst strength, indicating the research heat on this topic. Based on these results and after screening the titles and abstracts of these 685 papers, we concluded the hotspots in GIOP-related research in the recent 10 years as follows.

#### The role of BMSCs in the pathogenesis of GIOP

Bone marrow-derived mesenchymal stem cells (BMSCs) are multipotent stem cells with strong self-renewal ability and multidirectional differentiation potential. As precursors of some cell lineages including osteoblasts, chondrocytes, adipocytes, myoblasts, and fibroblasts, BMSCs are crucial in maintaining the dynamic homeostasis of bone tissue (26). Given that the formation of new bone is primarily dependent on osteoblasts that arise from BMSCs, impaired BMSCs may lead to imbalance between bone resorption and bone formation and sequentially cause osteoporosis. In recent years, the role of BMSCs in the pathogenesis of GIOP has attracted much attention. Some studies showed that low-dose GCs could promote cell viability and osteoblastic differentiation of BMSCs *in vivo* and *in vitro* (27, 28). On the contrary, high-dose GCs have been found to suppress osteoblast differentiation of BMSCs but promote them to differentiate into adipocytes (29, 30). In addition, high dosage of GCs can also induce an increase in apoptosis and cell death of BMSCs (31, 32). Therefore, protecting BMSCs from excessive GCs may be a promising direction for the prevention and treatment of GIOP in the future.

#### GIOP in postmenopausal women

It is well known that estrogen helps to maintain bone mass and strength in adults. Therefore, the deficiency of estrogen in postmenopausal women may cause imbalance in bone metabolism, gradually resulting in loss of bone mass and osteoporosis. As the life expectancy of global population increases, chronic diseases such as rheumatoid arthritis and asthma which require long-term GC treatment are becoming more and more common in postmenopausal women. When patients with osteoporosis were exposed to both postmenopausal and long-term GC use risk factors, the loss of bone mass would further accelerate and fracture risk could significantly increase (33). Scientists have paid more and more attention to this group in the past 10 years. One study found substantial cortical abnormalities and deteriorated trabecular microarchitectures in postmenopausal women using GCs, and the whole-bone stiffness was significantly decreased at their long bones (34). Some studies also established animal models to mimic the condition of postmenopausal women with GIOP, like rat (35), rabbit (36) and sheep (37, 38). Besides, the influence and treatment of GIOP on postmenopausal women have also been specifically concerned by experts in clinical studies (39–41).

#### Fracture risk in GIOP

The most important clinical significance of GIOP is in the occurrence of fractures. It was estimated that 30% to 50% of patients who had received long-term GC treatment would finally have an osteoporotic fracture, and the incidence of fracture grows with the duration of GC use (42, 43). Vertebral fracture is the most frequent GIOP-related fracture, while hip fracture is also not rare (42). Since fractures are usually highly burdensome for both the patient’s family and society, it is essential to identify the patients under GC treatment who are at high risk for fracture so as to provide interventions to them. Low bone mineral density (BMD) has been a well-known factor for causing fractures. But in fact, fractures often occur with relatively normal BMD values in GIOP patients, which complicates the identification of patients at risk for fracture (3, 44). Therefore, some new tools were developed to more accurately evaluate fracture risk. For instance, trabecular bone score (TBS) is an analytical tool for capturing information relating to trabecular microarchitecture, which has been proven to better predict fragility fractures than BMD values alone (45). There has been growing interests in the use of TBS in recent years, including in the GIOP field. The clinical utility of TBS for fracture risk assessment in GIOP patients has been supported by many clinical studies, and this tool is thought to be a prospective measurement complementary to BMD values in routine clinical evaluation (45, 46).

## Future research directions

From **Figure 6**, the hot topics varied over the years. The keywords “trial”, “randomized trial”, “men” and “controlled trial” were mainly distributed in the earlier years of the past decade, indicating that clinical study was the major trend of GIOP-related research at that time. However, the emerging keywords have gradually shifted to “marker”, “activation”, “oxidative stress”, “pathway”, “expression” and so on since 2017. The research focus in GIOP seems to have switched to molecular mechanism research. Based on the development of burst keywords and high-frequency and high-centrality keywords, the future research directions in this field could be summarized as follows.

(1) GCs, such as “dexamethasone”, could induce “oxidative stress” in bone microenvironment, “inhibition” of “osteogenesis”, and “apoptosis” of bone marrow-derived “mesenchymal stem cells”. Their underlying “mechanism” has been particularly explored in many *in vitro* studies in recent years, but *in vivo* ones were much fewer. Thus, further *in vivo* researches utilizing GIOP “models” are still needed in the future to validate *in vitro* experiment results and obtain new preclinical findings.

(2) The existing drugs for GIOP, typically “bisphosphonates”, work mainly *via* promoting the activity of osteoblasts and inhibition of “osteoclastogenesis”. However, these drugs still have many flaws related to safety issues, side effects and high costs (47). Many basic studies had found potential targets and drugs for novel clinical treatment on GIOP, and meanwhile explored the underlying signal “pathway” mechanism. This research trend seemed to be on the rise in recent years. In the future, experts may carry out more in-depth research of natural drugs (such as Chinese traditional herbs) or synthetic drugs which are potentially effective on treating GIOP, and elucidate their related mechanism and efficacy at the molecular, cellular, organ, and animal level. Moreover, high-quality clinical “trials” are also needed to validate the potential clinical utility of these novel treatments.

Our study has some limitations. First, only WoSCC database was chosen to search for papers due to limitations of CiteSpace software. Second, only published articles were included but meeting records, reviews, letters, and textbooks were excluded, which might cause omission bias. Third, only papers published in the English language were selected. Finally, only the articles published in the recent decade were retrieved in order to reflect the current state of this field. Therefore, a more comprehensive bibliometric study which utilizes a mix of databases and includes non-English papers without limitation of publication year could be conducted in the future to show the research trend of GIOP-related research in a wider view.

## Conclusion

In summary, this study identified the articles in the GIOP research field published between 2012 and 2021. We delineated their time distribution, highlighted the outstanding countries, institutions, authors, and journals, listed the top cited references, and conducted keyword analysis. This research field remained stable in recent years. The research hotspots in this field were majorly around the role of bone marrow-derived mesenchymal stem cells in the pathogenesis of GIOP, GIOP in postmenopausal women and fracture risk of GIOP. In the future, more basic and preclinical *in vivo* studies should be conducted in this field, and the potentially effective drugs for GIOP are worth further studies. Moreover, high-quality clinical trials should also be paid more attention. The results from this bibliometric study provided insight into the status and research trends in GIOP of the past decade, which could help researchers quickly determine the current hotspots and frontier trends and may encourage further practice in this field.

## Data availability statement

The original contributions presented in the study are included in the article/supplementary material. Further inquiries can be directed to the corresponding authors.

## Author contributions

BJ drafted and revised the manuscript. CF and CL drew all the pictures. CT reviewed and revised the manuscript. ZL contributed to the conception and design of the study. All authors contributed to the article and approved the submitted version.

## Funding

This work was supported by the National Natural Foundation of China (81902745, 82172500), Hunan Provincial Natural Science Foundation of China (2022JJ30843), the Science and Technology Development Fund Guided by Central Government (2021Szvup169), and Hunan Provincial Administration of Traditional Chinese Medicine Project (No. D2022117).

## Acknowledgments

The authors would like to thank Mr. Bolin Ren for helpful comments on the manuscript.

## Conflict of interest

The authors declare that the research was conducted in the absence of any commercial or financial relationships that could be construed as a potential conflict of interest.

## Publisher’s note

All claims expressed in this article are solely those of the authors and do not necessarily represent those of their affiliated organizations, or those of the publisher, the editors and the reviewers. Any product that may be evaluated in this article, or claim that may be made by its manufacturer, is not guaranteed or endorsed by the publisher.
